# Lithium Intercalation Chemistry in TaS_2_ Nanosheets for Lithium-Ion Batteries Anodes

**DOI:** 10.3390/nano15080626

**Published:** 2025-04-19

**Authors:** Xuelian Wang, Jin Bai, Xian Zhang, Xiaobo Shen, Zhengrong Xia, Haijun Yu

**Affiliations:** 1 School of Electronic Engineering, Huainan Normal University, Huainan 232038, China; wangxuelian@mail.ustc.edu.cn (X.W.); shenxb@hnnu.edu.cn (X.S.); zhengrongxia@hnnu.edu.cn (Z.X.); 2 Key Laboratory of Materials Physics, Institute of Solid State Physics, The Hefei Institutes of Physical Science (HFIPS), Chinese Academy of Sciences, Hefei 230031, China

**Keywords:** lithium-ion batteries, anode, TaS_2_ nanosheet, intercalation reaction mechanism, phase transition

## Abstract

Exploring novel two-dimensional layered transitional metal dichalcogenides and elucidating their reaction mechanism are critical to designing promising anode materials for lithium-ion batteries (LIBs). Herein, a novel layered TaS_2_ nanosheet was obtained via a typical solid-phase reaction method followed by a simple ball-milling treatment, and first explored experimentally as an anode for LIBs. The TaS_2_ nanosheet anode delivered an excellent cycling stability, with 234.6 mAh g^−1^ after 500 cycles at 1 A g^−1^. The optimized performance could be attributed to the large interlayer spacing, high conductivity, and reduced size of the TaS_2_ nanosheet, which effectively alleviated the volume change during the reaction process and accelerated the Li^+^ or e^−^ transport. Especially, the TaS_2_ nanosheet anode presented an unusual intercalation reaction mechanism, accompanied with a reversible phase transition from the 2H to the 1T phase during the first de-lithiation process, which is evidenced by the multiple ex situ characterizations, further revealing the enhanced electrochemical performance results from the 1T phase with the larger interlayer spacing and higher electrical conductivity. This work provides a novel insight into the intercalation reaction mechanism of TaS_2_, which shows potential in high-performance LIBs.

## 1. Introduction

Lithium-ion batteries (LIBs) have been widely applied in the various portable electronic devices and electric vehicles due to their high energy density and long cycling lifespan [1,2,3]. However, the present LIBs based on the graphite anode cannot meet the increasing requirements for high-capacity and highly safe application scenarios due to the low theoretical capacity (372 mAh g^−1^) and potential safety issues in the graphite anode [4,5]. Thus, searching for more suitable anode materials for high-performance LIBs is now urgent.

Transitional metal dichalcogenides (TMDs), having the chemical formula MX_2_ (M = Mo, W, etc.; X = S, Se, and Te), with an interlayer coupled by weak van der Waals force, have received widespread attention in various research fields, such as energy storage, catalysis, and electronics, due to their rich physical and chemical properties [6,7]. In recent years, more and more studies have suggested that TMDs are a class of promising anode materials for LIBs because of their high theoretical capacities and large interlayer spacing, facilitating efficient lithium storage and transport [8,9]. At present, many studies have been devoted to the conversion reaction mechanism of MX_2_ anodes, for example, MoS_2_. However, they still suffered a large volume change during cycling, bringing structural collapse and poor electrical conductivity, thus causing an inferior rate capability when acting as an anode for LIBs [10,11]. Regarding these issues, researchers have performed many studies to modify the electrochemical performance of this kind of anode. The main modification strategy is the introduction of carbonaceous materials (e.g., graphene or carbon nanotubes) to construct MX_2_-based composites [12,13]. Nevertheless, this will undoubtedly increase the cost of the manufacturing process, which makes it potentially difficult to change the nature of the poor conductivity and the large volume change, thus leading to an unsatisfactory long cycling stability and high-rate performance. Therefore, exploring novel MX_2_ materials with intrinsic intercalation reaction mechanisms and high conductivity as promising anode materials for high-performance LIBs has significant research value. Previous theoretical calculations and experimental results indicate that layered TaS_2_ material has been widely studied in the condensed matter physics fields due to its rich physical properties, showing large interlayer spacing and room-temperature metallic conductivity behavior [14]. Wu et al. pioneered the theoretical study on TaS_2_ as an anode for LIBs via density functional theory (DFT) calculations, presenting TaS_2_ as having metallic conductivity behavior around the Fermi level, as well as a smaller Young’s modulus than MoS_2_, thus showing it to be the ideal candidate for high-performance LIB anodes [15]. Qiao et al. performed a theoretical study on the MoS_2_@TaS_2_ vdW heterostructure for LIB anodes, exhibiting a high theoretical capacity of 589 mAh g^−1^ [16]. Mao et al. developed a zero-strain 2D TaSe_2_ as an anode for LIBs, displaying high areal and gravimetric specific capacities [17]. Conversely, experimental studies on TaS_2_ as an anode for LIBs have rarely been reported.

In this work, a TaS_2_ nanosheet material was successfully synthesized by a typical solid-phase reaction, followed by a facile ball-milling process. The obtained TaS_2_ exhibited a 2H phase structure and a reduced nanosheet size. Owing to the large interlayer spacing, high intrinsic conductivity, and reduced nanosheet size, the TaS_2_ nanosheet anode displayed an excellent electrochemical performance with a high reversible capacity of 484.9 mAh g^−1^ after 300 cycles at 0.1 A g^−1^, and 234.6 mAh g^−1^ after 500 cycles at 1 A g^−1^. Furthermore, it presented an unusual intercalation reaction mechanism with reversible phase transition from the 2H to the 1T phase during the first charging process (for the concepts of the 2H and 1T phases, see Note S1), thereby strongly clarifying the origin of the increasing capacity during cycling and the long cycling stability.

## 2. Results and Discussion

**Figure 1:**
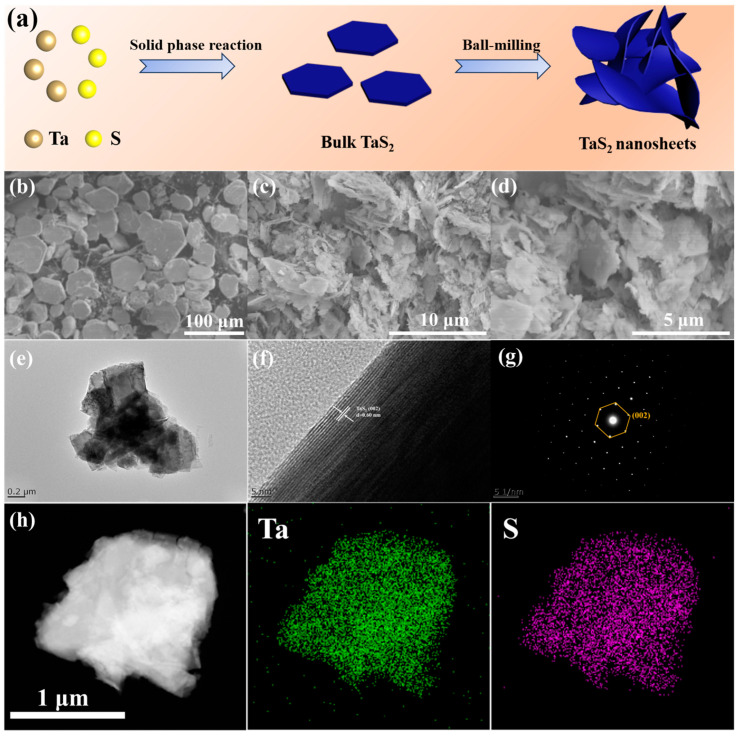
(**a**) Schematic illustration of the synthesis process of the b-TaS_2_ and n-TaS_2_ samples. SEM images of (**b**) b-TaS_2_ and (**c**,**d**) n-TaS_2_ at different magnifications. (**e**) TEM image, (**f**) HRTEM image, (**g**) SAED image, and (**h**) EDS-mapping image of the n-TaS_2_ samples.

Figure 1a shows a schematic illustration of the synthesis process of the TaS_2_ nanosheets (n-TaS_2_) and bulk TaS_2_ (b-TaS_2_) samples. Figure 1b and Figure S1a,b show the SEM images of the b-TaS_2_ sample. A hexagonal disk-shaped morphology is easily observed, and the corresponding lateral size and thickness are about 50–100 μm and 5 μm, respectively. For the n-TaS_2_ sample, a nanosheet morphology is exhibited from the SEM images in Figure 1c,d, resulting from the exfoliation of the b-TaS_2_ sample by the ball milling. The high-magnification SEM image in Figure 1d further displays that the lateral size of the n-TaS_2_ sample is about 1–2 μm, with a thickness of about 100 nm, indicating that the ball-milling process significantly decreased the size of the TaS_2_ material. Figure 1e and Figure S2 show the TEM images of the n-TaS_2_ sample, further proving the reduced lateral size of about 1 μm and a thickness of about 100 nm after the ball-milling exfoliation process, which could be beneficial to the enhanced lithium storage performance, as is discussed below. The HRTEM image of the n-TaS_2_ sample is displayed in Figure 1f. The interlayer spacing of about 0.60 nm corresponds to the characteristic (002) plane of the 2H phase in TaS_2_. The selected area electron diffraction (SAED), shown in Figure 1g, indicates that the individual nanosheets in the n-TaS_2_ sample still retain the diffraction spots characteristic of the monocrystal, although whole sample has a powder character. Figure 1h exhibits the EDS-mapping images of the n-TaS_2_ sample, and Ta and S elements with an atomic ratio near 1:2 are homogeneously distributed in the whole sample, suggesting a reliable TaS_2_ structure.

**Figure 2:**
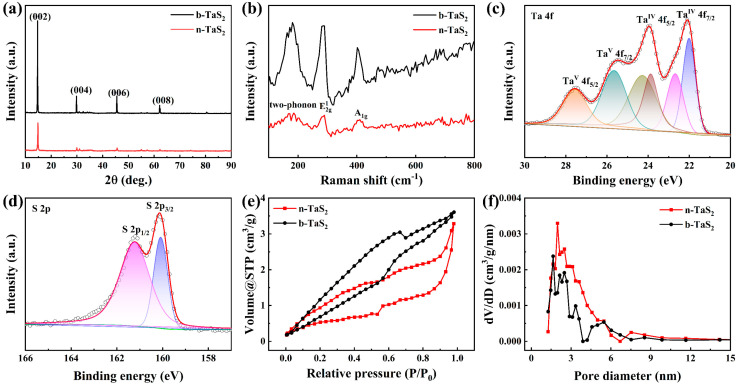
(**a**) XRD patterns and (**b**) Raman spectra of the b-TaS_2_ and n-TaS_2_ samples. High-resolution (**c**) Ta 4f XPS spectrum and (**d**) S 2p XPS spectrum of the n-TaS_2_ sample. (**e**) Isotherm N_2_ adsorption–desorption curves and (**f**) pore diameter distributions of the b-TaS_2_ and n-TaS_2_ samples.

Figure 2a shows the XRD patterns of the b-TaS_2_ and n-TaS_2_ samples. All of the diffraction peaks can be matched with the 2H phase of TaS_2_ with a hexagonal structure (JCPDS Card No. 80-0685) [18], and no impurity phases can be found. They all exhibit a crystal face orientation of (00*l*) planes, and only the peak intensity of the (002) plane is obviously stronger than the other (00*l*) planes. After suffering the ball-milling process, the lattice parameters become slightly smaller, the full width at half maximum (FWHM) value of the diffraction peaks become larger, and the peak intensities become weaker for the n-TaS_2_ sample, resulting in the crystallite size becoming smaller, as shown in Table S1, which agrees well with the above SEM and TEM analyses. The Raman spectra of the b-TaS_2_ and n-TaS_2_ samples are provided in Figure 2b. They show similar vibration peaks located at about 180, 286, and 408 cm^−1^ [19], with weaker vibration peaks in the n-TaS_2_ sample after the ball-milling process. Meanwhile, the Raman peak at about 180 cm^−1^ originates from the two-phonon scattering mode, which is common to the 2H TaS_2_ structure with a charge density wave (CDW) phase transition. The Raman peak at about 286 cm^−1^ is indexed to the $E_{2g}^{1}$ mode, which is attributed to the in-plane vibration of the 2H TaS_2_. The Raman peak at about 408 cm^−1^ corresponds to the A_1g_ mode, which is assigned to the out-of-plane vibration of the 2H TaS_2_. This indicates that the n-TaS_2_ retains the original structural characteristics of the b-TaS_2_, which is also consistent with the XRD analysis. The survey XPS spectrum provides the coexistence of Ta and S elements in the n-TaS_2_ sample, as shown in Figure S3. The high-resolution XPS spectrum of Ta 4f, shown in Figure 2c, displays the two main peaks at about 22.0 and 24.3 eV, corresponding to Ta 4f_7/2_ and Ta 4f_5/2_ of Ta^4+^, respectively, with the two satellite peaks of 22.7 and 23.8 eV [20]. Additionally, the additional two peaks at about 25.6 and 27.5 eV are attributed to Ta 4f_7/2_ and Ta 4f_5/2_ of Ta^5+^, respectively, which could have originated from the little oxidation on the surface of the sample [21]. Figure 2d exhibits the high-resolution XPS spectrum of the S 2p with two obvious peaks at 160.1 and 161.2 eV, which are assigned to S 2p_3/2_ and S 2p_1/2_ of S^2−^, respectively [22]. The above elements and valance state analyses further confirm the reliable structure of n-TaS_2_, which also aligns with the above EDS analysis. The isothermal N_2_ adsorption–desorption curves of the b-TaS_2_ and n-TaS_2_ samples are shown in Figure 2e, showing a mesoporous character. The corresponding specific areas were calculated to be 7.2 and 10.9 m^2^/g using the BET method. Furthermore, the pore diameter distributions of both samples are also provided in Figure 2f, which displays that the n-TaS_2_ sample has the larger and richer mesopores ranging from 2 to 10 nm, obtained via the BJH method. The enhanced specific area and mesoporous distribution in the n-TaS_2_ sample are attributed to the reduced sheet sizes brought from the ball-milling exfoliation process, which could promote the fast transfer of Li^+^, as is discussed below.

**Figure 3:**
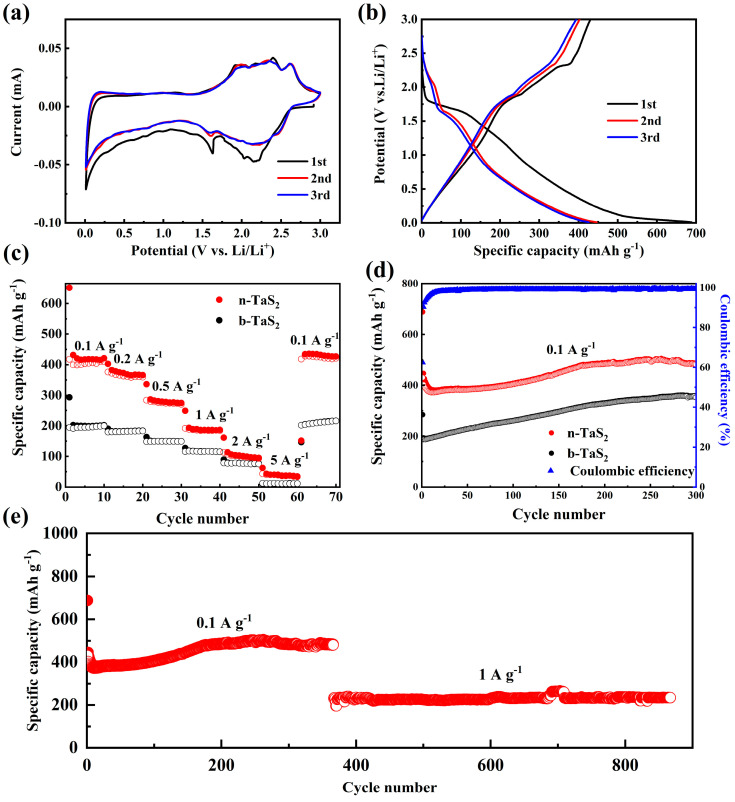
First three cycles of the (**a**) CV curve at 0.1 mV s^−1^ and (**b**) GCD curves at 0.1 A g^−1^ of the n-TaS_2_ electrode. (**c**) Rate capability and (**d**) cycling performance at 0.1 A g^−1^ of the n-TaS_2_ and b-TaS_2_ electrodes. (**e**) Long-term cycling performance at 0.1 and 1 A g^−1^.

The electrochemical performance of b-TaS_2_ and n-TaS_2_ as the anodes for LIBs have been evaluated via assembling CR2032 coin half-cells with lithium foil as the counter electrode. The initial three cyclic voltammetry (CV) curves at a scanning rate of 0.1 mV s^−1^ in the voltage range from 0.01 to 3 V (vs. Li/Li^+^) for the b-TaS_2_ and n-TaS_2_ electrodes are shown in Figure 3a and Figure S4, respectively. For the b-TaS_2_ electrode, the reduction peak at about 1.61 V could be assigned to the Li^+^ insertion into the interlayer of the b-TaS_2_ electrode, forming Li*_x_*TaS_2_. In the subsequent cycles, two oxidation peaks at about 1.90 and 2.34 V can be observed, which could be assigned to the gradual de-insertion process of Li^+^ in the TaS_2_ matrix. For the n-TaS_2_ electrode, there are the two reduction peaks at about 2.16 and 1.63 V, as shown in Figure 3a, which could be attributed to the gradual Li^+^ insertion into the interlayer of the n-TaS_2_ electrode. In the subsequent scanning of the n-TaS_2_ electrode, two oxidation peaks at about 1.96 and 2.37 V could correspond to the gradual de-insertion process, like that observed for the b-TaS_2_ electrode, and an additional peak at about 2.61 V could result from the deep de-lithiation process accompanied with the complex phase transformation, which will be further clarified via the ex situ measurements, as is discussed below. In the subsequent scanning, the redox peaks of the n-TaS_2_ electrode well overlap, indicating excellent electrochemical reversibility. The first three cycles of the galvanostatic charge/discharge (GCD) profiles of the n-TaS_2_ and b-TaS_2_ electrodes at 0.1 A g^−1^ are shown in Figure 3b and Figure S5, respectively. The n-TaS_2_ electrode delivered initial discharge and charge capacities of 688.5 and 430.1 mAh g^−1^, which are greatly higher than that of the b-TaS_2_ electrode (293.1 and 194.3 mAh g^−1^). The well-overlapped GCD profiles in the subsequent cycles for both of the electrodes suggest the strong cycling reversibility. Figure 3c shows the rate capability comparison of both electrodes. The n-TaS_2_ electrode delivered the higher capacities of 418.1, 374.4, 280.7, 188.9, 103.7, and 40.2 mAh g^−1^ at current densities of 0.1, 0.2, 0.5, 1, 2, and 5 A g^−1^, respectively, which are greatly higher than those of the b-TaS_2_ electrode. When returned to 0.1 A g^−1^, the capacity of the n-TaS_2_ electrode increased to 425.2 mAh g^−1^, indicating the former electrochemical activation process. It can be observed that only the capacity of 11.1 mAh g^−1^ can be delivered at 5 A g^−1^ for the b-TaS_2_ electrode, suggesting the enhanced charge transfer ability of the n-TaS_2_ electrode, which could be related to the smaller nanosheet size after the ball-milling process. The cycling performance comparison of both electrodes is displayed in Figure 3d. Both electrodes exhibit a similar trend of a capacity increase, while the n-TaS_2_ electrode delivered the higher reversible capacity of 484.9 mAh g^−1^ compared to that of the b-TaS_2_ electrode (352.4 mAh g^−1^) after 300 cycles at 0.1 A g^−1^. The reason for the increasing capacity with the cycling could be attributed to the complex structural evolution during the insertion/de-insertion processes, as is discussed below. To further confirm the long-term cycling stability of the n-TaS_2_ electrode, Figure 3e shows the longer cycling performance at 0.1 and 1 A g^−1^. It can be found that the capacity remains stable, with about 484 mAh g^−1^ after 366 cycles, and maintains a nearly constant capacity of 234.6 mAh g^−1^ with a capacity retention of 101.1% after 866 cycles at 1 A g^−1^. To clarify the origin of the dynamics for the enhanced rate capability, we performed electrochemical impedance spectra (EIS) measurements on both electrodes, as shown in Figure S6. The result indicates that the n-TaS_2_ has a smaller charge transfer resistance (R_ct_) and larger linear slope than the b-TaS_2_, suggesting an enhanced charge transfer ability and promoted lithium ion diffusion kinetics.

**Figure 4:**
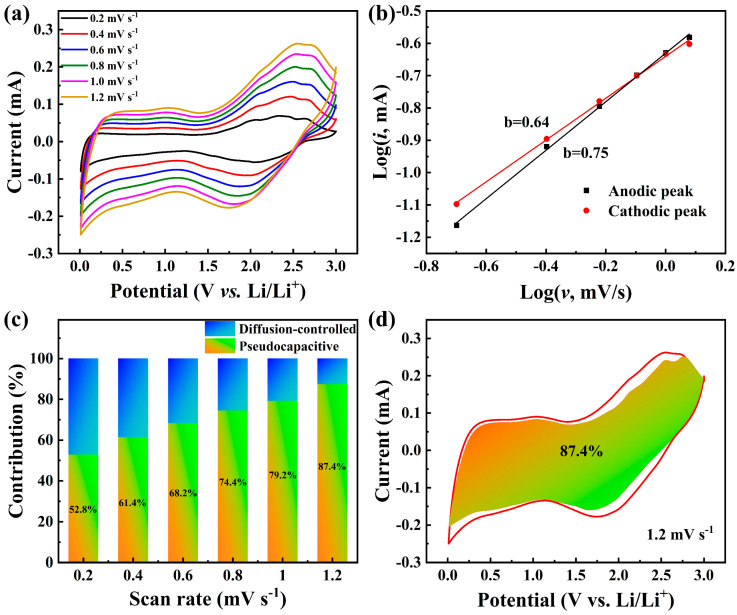
(**a**) CV curves at various scanning rates, (**b**) linear fitting results of the Log(*i*)-Log(*v*) relationship, (**c**) the pseudocapacitive contribution ratios at different scanning rates, and (**d**) a typical pseudocapacitive fitting result at 1.2 mV s^−1^ for the n-TaS_2_ electrode.

To further understand the internal kinetic behavior for the enhanced lithium storage performance, the CV measurements of the n-TaS_2_ electrode at the different scanning rates ranging from 0.2 to 1.2 mV s^−1^ were performed and are shown in Figure 4a. Among them, the relationship between the peak current (*i*) and scanning rate (*v*) obeys the following Equation (1) [23]:

*i* = a*v*^b^ (1)

where b = 0.5 represents diffusion-controlled process, b = 1 represents pseudocapacitive process, and 0.5 < b < 1 represents the mixed-charge storage behavior. The linear fitting results of Log(*i*)-Log(*v*) at the anodic and cathodic peaks (at about 1.7 and 2.4 V) are plotted in Figure 4b. The calculated b = 0.75 at the anodic peak and b = 0.64 at the cathodic peak indicate that the charge storage behavior of the n-TaS_2_ electrode is commonly governed pseudocapacitive and diffusion-controlled processes. To further quantify the contribution of the charge storage behavior to the capacity, the following Equation (2) was employed to calculate the respective contribution [24]:

*i* = k_1_*v* + k_2_*v*^1/2^ (2)

where k_1_*v* represents the pseudocapacitive contribution and k_2_*v*^1/2^ stands for the diffusion-controlled contribution. The calculated pseudocapacitive contributions at 0.2–1.2 mV s^−1^ are 52.8%, 61.4%, 68.2%, 74.4%, 79.2%, and 87.4%, respectively, as shown in Figure 4c. Especially at 1.2 mV s^−1^, a typical fitting result (gradient filling region) is shown in Figure 4d, which displays the high pseudocapacitive contribution ratio of 87.4%, indicating that the reduced nanosheet size in the n-TaS_2_ electrode significantly promotes the fast Li^+^ transport ability, especially at high current densities.

**Figure 5:**
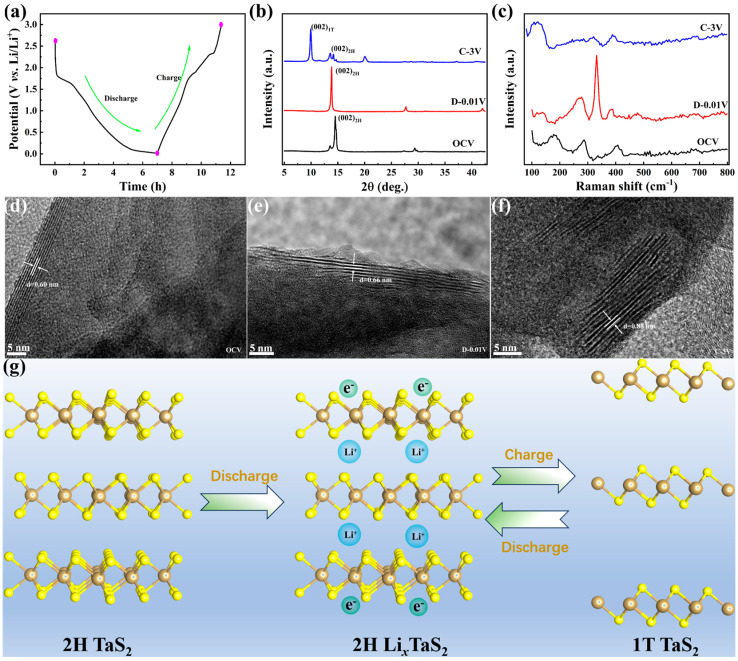
(**a**) The selected studied potentials at the first GCD curves, (**b**) ex situ XRD patterns, (**c**) ex situ Raman spectra, (**d**–**f**) ex situ HRTEM images, and (**g**) schematic diagram of the proposed reaction mechanism of the n-TaS_2_ electrode.

To further reveal the charge reaction mechanism of the n-TaS_2_ electrode, the multiple ex situ measurements, including ex situ XRD, ex situ Raman, and ex situ TEM at the first discharge/charge processes, were carried out and are shown in Figure 5. Figure 5a shows the first GCD profile of the n-TaS_2_ electrode, and the corresponding ex situ XRD patterns at the selected charging/discharging states are shown in Figure 5b. At the open circuit voltage (OCV) state, the n-TaS_2_ electrode presents similar diffraction peaks to the powder sample, corresponding to the 2H phase TaS_2_ with a typical characteristic (002) peak. When discharged to 0.01 V, the (002) peak shows an obvious left shift, indicating the Li^+^ intercalation into the n-TaS_2_, accompanied with the increased interlayer spacing. When charged to 3 V, an unusual result is displayed, where the (002) peak does not simply shift back to the higher angle, but a structural transformation from the 2H to the 1T phase occurs. To further prove the unusual the electrochemical reaction mechanism, the ex situ Raman spectra of the n-TaS_2_ electrode are shown in Figure 5c. At the OCV state, the Raman spectrum still shows similar vibration modes with the powder sample. As it is discharged to 0.01 V, the $E_{2g}^{1}$ mode appears with an evident blue shift, and its peak intensity becomes stronger, suggesting the intercalation of Li^+^ could greatly affect the in-plane vibration of the n-TaS_2_. As it is charged to 3 V, a similar phenomenon to the ex situ XRD pattern appears, showing that the Raman spectrum does not return to the original vibration modes, but rather displays a set of new vibration peaks. The peak at about 100 cm^−1^ is assigned to the vibration mode of the 1T phase, while the peaks at 318.2 and 392.5 cm^−1^ are attributed to the $E_{2g}^{1}$ and A_1g_ modes of the 2H phase, indicating the phase transformation behavior during the initial charging process. Ex situ TEM measurements were also performed and are presented in Figure 5d–f. The HRTEM image of the n-TaS_2_ electrode displays interlayer spacing of about 0.60 nm, which is similar with the powder sample at the OCV state, as shown in Figure 5d. When discharged to 0.01 V, the interlayer spacing increases to 0.66 nm due to the intercalation of lithium ions, as shown in Figure 5e. Moreover, the HRTEM image, depicted in Figure S7, indicates that the in-plane structure of the n-TaS_2_ electrode retains the 2H phase structure when Li^+^ inserts into the interlayer. Figure 5f presents that the interlayer spacing becomes larger, at about 0.88 nm when charged to 3 V. This unusual phenomenon is further clarified by the in-plane HRTEM image of the n-TaS_2_ electrode, shown in Figure S8, which displays the co-existence of the 1T and 2H phases in the n-TaS_2_, confirming the phase transformation mechanism during the first charging process. The above analysis is also similar with the XRD and Raman results. To intuitively demonstrate the charge storage mechanism of the n-TaS_2_ electrode, a schematical illustration is depicted in Figure 5g. Regarding to the reaction process, we provide the possible reaction equations, as shown in Note S2. In detail, the lithium ions insert into the interlayer of the TaS_2_ during the initial discharging process, forming Li*_x_*TaS_2_. In the subsequent charging process, the lithium ions remove from the interlayer, accompanied by the structural transformation from the 2H to the 1T phase. Subsequently, the n-TaS_2_ electrode performs reversible phase transition behavior in the subsequent cycles based on reversible intercalation/de-intercalation processes. The phase transition behavior between the 2H and 1T phases could be attributed to the large amount of lithium ion removal during the charging process caused the local structural distortion, inducing the formation of the 1T phase in the n-TaS_2_ anode. Owing to the larger interlayer spacing and higher conductivity of the 1T phase, the n-TaS_2_ anode presents a gradually increasing capacity with cycles and long-term cycling stability. Whereas, as the lithium ions re-embedded into the interlayer of the 1T TaS_2_, the n-TaS_2_ returned back to the 2H phase, driven by the minimum thermodynamic energy principle of the system.

## 3. Conclusions

In summary, TaS_2_ nanosheets were prepared via a typical solid-phase reaction method followed by a simple ball-milling treatment. More importantly, we performed a comprehensive experimental study on the TaS_2_ nanosheet as an anode for LIBs for the first time. The morphological and structural characterizations suggest that the TaS_2_ nanosheet sample has a 2H phase structure as well as reduced nanosheet size. When used as an anode for LIBs, it delivered a high reversible capacity of 484.9 mAh g^−1^ after 300 cycles at 0.1 A g^−1^, and 234.6 mAh g^−1^ after 500 cycles at 1 A g^−1^, displaying an excellent cycling performance. The remarkable electrochemical performance could be attributed to the reduced nanosheet size accelerating the charge transfer and promoting the pseudocapacitive adsorption behavior. More importantly, the unusual intercalation reaction mechanism in the TaS_2_ nanosheet electrode revealed by the ex situ XRD, ex situ Raman, and ex situ TEM measurements shows reversible phase transition behavior from the 2H to the 1T phase during the first charging process, which could contribute to the increasing capacity with cycles and long-term cycling stability. Thus, the novel TaS_2_ nanosheet could be a potential anode material for high-performance LIBs.

## Data Availability

Data are contained within the article and Supplementary Materials.

## Supplementary Materials

The following supporting information can be downloaded at: https://www.mdpi.com/article/10.3390/nano15080626/s1, Figure S1. The high-magnification SEM images of the b-TaS_2_ sample. Figure S2. The TEM image of the n-TaS_2_ sample. Figure S3. The survey XPS spectrum of the n-TaS_2_ sample. Figure S4. The first three cycles CV curves of the b-TaS_2_ electrode at 0.1 mV s^−1^. Figure S5. The first three cycles GCD curves of the b-TaS_2_ electrode at 0.1 A g^−1^. Figure S6. The EIS comparison of n-TaS_2_ and b-TaS_2_ electrodes. Figure S7. The HRTEM image of the n-TaS_2_ electrode at the fully discharged state of 0.01 V. Figure S8. The HRTEM image of the n-TaS_2_ electrode at the fully charged state of 3 V. Table S1. The crystal structure parameters of n-TaS_2_ and b-TaS_2_ samples. Note S1. Explanations of 2H and 1T phases. Note S2. The possible electrochemical reaction equations of n-TaS2 electrode.
