# Supplementary material for: Lithium Intercalation Chemistry in TaS2 Nanosheets for Lithium-Ion Batteries Anodes
Source: Nanomaterials (Basel). 2025 Apr 19;15(8):626. doi: 10.3390/nano15080626 (PMC12029614; doi:10.3390/nano15080626)
Supplement: Supplementary file 1 [file nanomaterials-15-00626-s001.zip › nanomaterials-3578067-supplementary.pdf]

Lithium Intercalation Chemistry in TaS_2 Nanosheets for Lithium-Ion Batteries Anodes

Xuelian Wang [1,2], Jin Bai [2,*], Xian Zhang [1,*], Xiaobo Shen [1], Zhengrong Xia [1] and Haijun Yu [1,*]

1 School of Electronic Engineering, Huainan Normal University,

Huainan 232038, China

2 Key Laboratory of Materials Physics, Institute of Solid State Physics, The Hefei Institutes of Physical Science (HFIPS), Chinese Academy of Sciences,

Hefei 230031, China

***Corresponding Authors**

jbai@issp.ac.cn (J.B.); zhangxian035@163.com (X.Z.); haijun20030@163.com (H.Y.)

Experimental

Chemicals

The Ta (AR, 99.9%) and S (AR, 99.99%) powders were purchased by Shanghai Aladdin Biochemical Technology Co., Ltd., without further purification.

Materials synthesis

The bulk TaS_2 was synthesized the following solid phase reaction process. Ta and S powders according to the stoichiometric ratio of 1:2 were mixed and sealed in an evacuated quartz tube. The mixture was heated up to 1173 K and then slowly cooled down to 673 K at a rate of 4 K/h. Next, the furnace was rapidly cooled down to room temperature to obtain the bulk TaS_2 sample, denoted as b-TaS_2. Subsequently, a certain amount of bulk TaS_2 powder was encapsulated in a ball-milling tank in the glove box filled by high-purity Ar atmosphere, and then treated via a ball-milling at a rotating speed of 400 r/min for 12 h to achieve the targeted TaS_2 nanosheets sample, denoted as n-TaS_2.

Materials characterizations

Powder X-ray diffraction (XRD) with Cu Kα radiation in the 2θ range from 10° to 90° was employed to characterize the structure and phase purity of as-prepared samples. Morphologies and grains size of all the samples were observed by using the field-emission scanning electron microscopy (FE-SEM, SU8020, HITACHI). Microstructure information was studied by the transmission electron microscopy (TEM, Tecnai G2F20) and high-resolution transmission electron microscopy (HRTEM). Element components were analyzed via X-ray energy dispersive spectra (EDS) attached to TEM. Specific

surface areas and pore diameter distributions were obtained from the N_2 adsorption and desorption measurements (Autosorb-iQ-Cx) at 77 K by the Bruauer-Emmett-Teller (BET) methods. X-ray photoelectron spectra (XPS) were performed on a Thermo Scientific ESCALAB 250 with Al Kα as the excitation source.

Electrochemical measurements

The batteries tests were implemented using a CR2032 coin-type cell. The working electrodes were fabricated by coating a slurry consisting of active material, Super P and CMC with a weight ratio of 8:1:1 in a certain amount of deionized water onto a copper foil. The slurry was dried in vacuum oven at 80 °C overnight. The coin cells were assembled in a Mbraun glove box filled with high-purity argon using the lithium metal foils as the counter electrode, Celgard 2325 porous polypropylene film as a separator and 1 M $LiPF_6$ in the organic solution of EC: EMC: DMC =1:1:1 as electrolyte. The galvanostatic charge and discharge tests were performed in a voltage range of 0.01-3.0 V (vs. Li/Li^+) at room temperature by a Land battery testing instrument. The cyclic voltammetry (CV) and electrochemical impedance spectroscopy (EIS) measurements were performed on an electrochemical workstation at room temperature. The potential window used in the CV tests is at 0.01-3 V (vs. Li/Li^+) at a scan rate of 0.1-1.2 mV s^{-1}. The EIS was measured in the frequency range between 0.01 Hz and100 kHz.

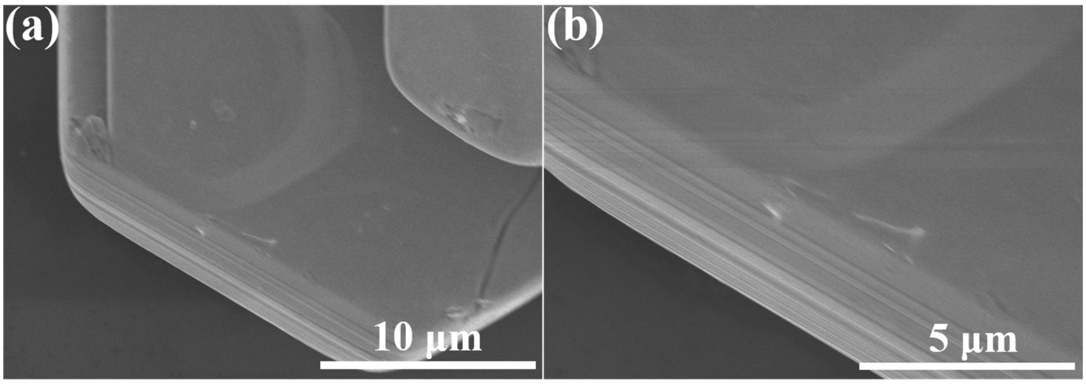

Figure S1. The high-magnification SEM images of the b-TaS_2 sample.

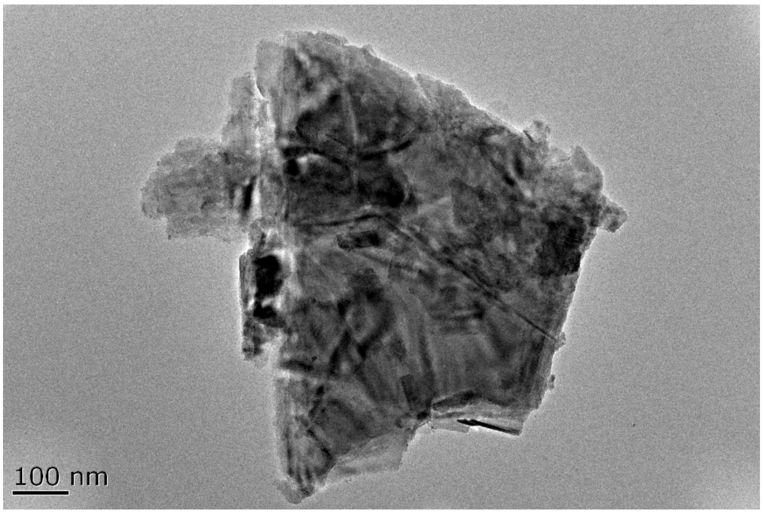

Figure S2. The TEM image of the n-TaS_2 sample.

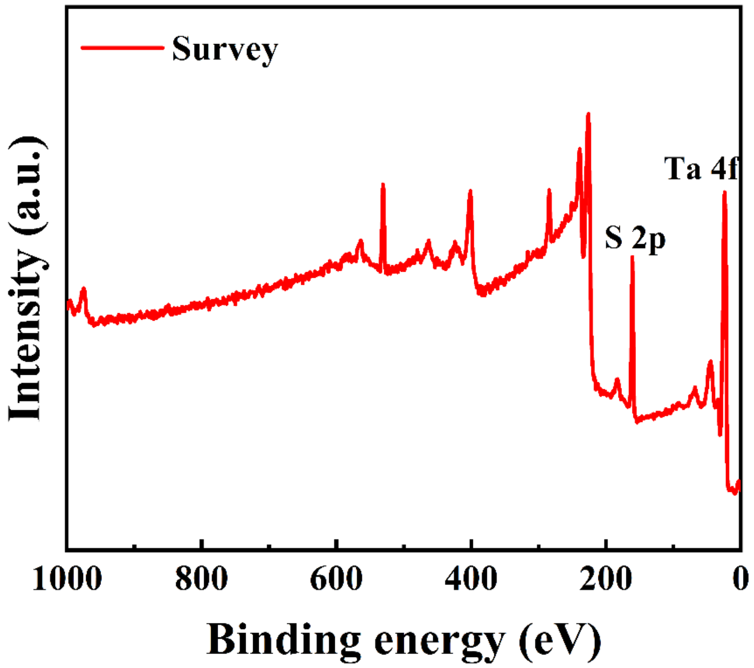

Figure S3. The survey XPS spectrum of the n-TaS_2 sample.

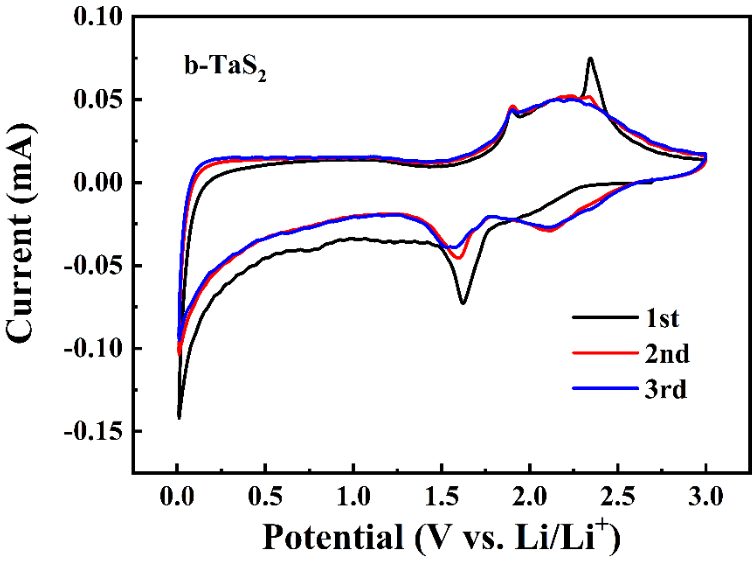

Figure S4. The first three cycles CV curves of the b-TaS_2 electrode at 0.1 mV s^{-1}.

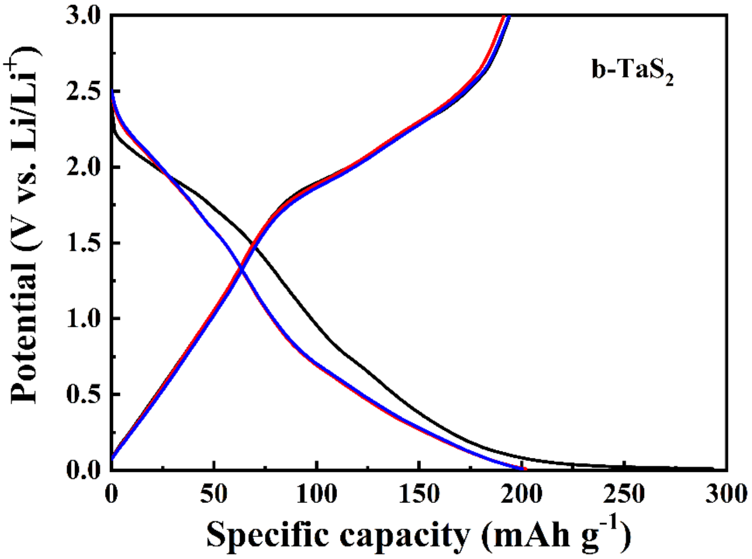

Figure S5. The first three cycles GCD curves of the b-TaS_2 electrode at 0.1 A g^{-1}.

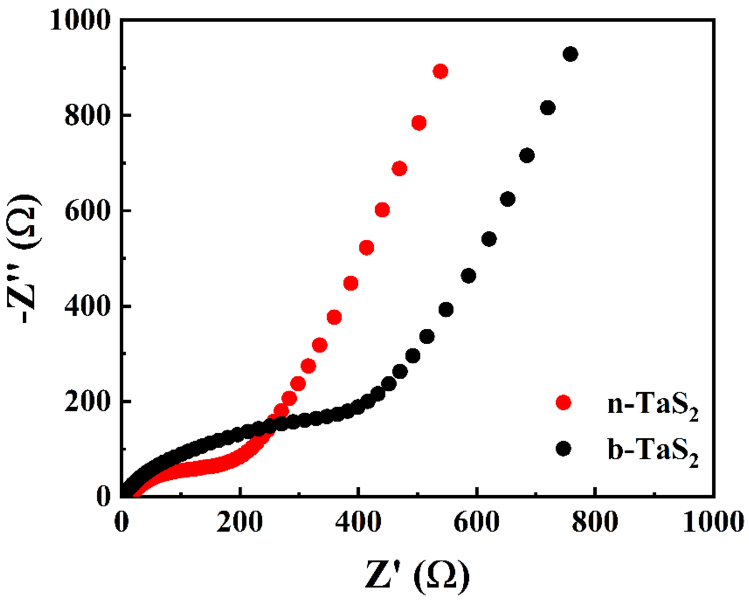

Figure S6. The EIS comparison of n-TaS_2 and b-TaS_2 electrodes.

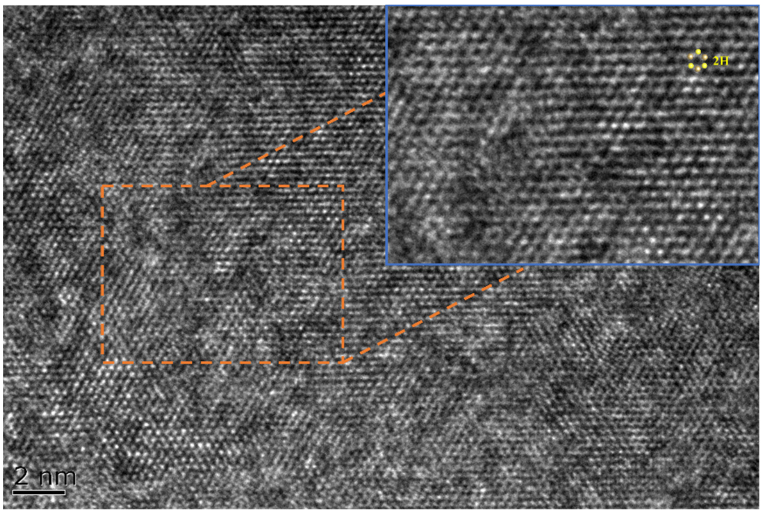

Figure S7. The HRTEM image of the n-TaS_2 electrode at the fully discharged state of 0.01 V.

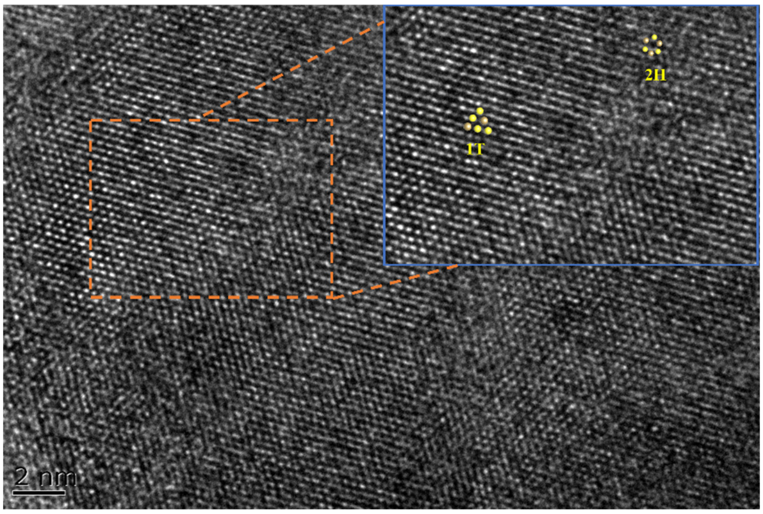

Figure S8. The HRTEM image of the n-TaS_2 electrode at the fully charged state of 3 V.

Table S1 The crystal structure parameters of n-TaS_2 and b-TaS_2 samples.

Samples	Space group	α	β	γ	a (Å)	b (Å)	c (Å)	FWHM (2θ/°)	Crystallite size (nm)
n-TaS_2	P63/mmc	90°	90°	120°	3.329	3.329	11.910	0.130	61.7
b-TaS_2					3.330	3.330	11.988	0.097	82.2

Note S1

Explanations of 2H and 1T phases:

Regarding to the layered transitional metal dichalcogenides (chemical formula: MX_2, M=Mo, Ta, etc.; X=S, Se, Te), they own several polymorphs, mainly including 2H and 1T, standing for hexagonal and trigonal phases, respectively. According to different coordination and stacking period, 2H phase owns trigonal prismatic coordination structure between M and X atoms, and presents two-layer stacking period, while 1T phase has octahedral coordination structure, and shows single-layer stacking period.

Note S2

The possible electrochemical reaction equations of n-TaS_2 electrode:

$$2H\ TaS_2 + xLi^+ + xe^- \rightarrow 2H\ Li_xTaS_2 \quad (S1)$$

$$2H\ Li_xTaS_2 - xLi^+ - xe^- \leftrightarrow 1T\ TaS_2 \quad (S2)$$
